# Factors associated with information literacy of nursing undergraduates in China

**DOI:** 10.1186/s12912-022-00855-9

**Published:** 2022-04-07

**Authors:** Xing Li, Jia-Yi Zhang, Yan-Xue Zheng, Ya-Xi Wang, Wen-Nv Hao

**Affiliations:** 1 Thyroid Breast Surgery, Affiliated Hospital of Inner Mongolia Medical University, Hohhot, China; 2 School of Nursing, Inner Mongolia Medical University, Hohhot, Inner Mongolia China; 3 Department of Emergency, Affiliated Hospital of Inner Mongolia Medical University, Huimin District, No. 1 Tongdao North Road, Hohhot, 010050 Inner Mongolia China

**Keywords:** Information literacy, Nursing undergraduates

## Abstract

**Objective:**

Information literacy is an important foundation for evidence-based nursing practice. Quantitative studies using validated questionnaires on information literacy of nursing undergraduates in China are rare. The students’ baseline information literacy must be evaluated before exploring ways to improve their level of information literacy. This study aimed to investigate the factors potentially involved in the information literacy of nursing undergraduates.

**Methods:**

In this cross-sectional study, male and female nursing undergraduates (*n* = 710) from Inner Mongolia, China were included in the final sample. The Information Literacy Competency Scale of the Applied Undergraduate Student (ILCSAUS) was used for evaluation. Multivariate stepwise linear regression analysis was performed to assess the association between various factors associated with information literacy.

**Results:**

The students’ information literacy score was 105.00 (94.00–119.00). The highest score in the four dimensions was information awareness dimension, and the average score was 4.00 (3.80–4.40). Multivariable stepwise linear regression analysis showed that received training in medical statistics, received training in literature retrieval and utilization, and birth place were independently associated with information literacy scores (*p*  < 0.05).

**Conclusions:**

Results indicate that learning about medical statistics, literature retrieval and utilization, and paying attention to students born in countries and towns can help improve information literacy in the nursing undergraduates.

## Introduction

Information literacy has become a major focus in recent times. Much of the discussion around this issue has arisen in recognition that we have entered an age where "The quality and quantity of information needed to function effectively in society and the workplace continues to increase. Individuals must be able to master rapidly changing information technology and possess the information literacy skills to act independently in this information rich environment" [1]. Information literacy as a concept focuses on a person's ability to interact with information and the cognitive and behavioural processes that are involved [2]. Information literacy is the term used to describe a number of initiatives in higher education that seek to meet the broad demands of the information society [3]. Information literacy is an important foundation for evidence-based nursing practice [4]. As nursing informatics expert Diane Skiba has noted, information literacy and informatics are both integral to the success of EBP [5].

Nurses in the 21st-century are expected to be data and information literate and proficient in data management. Nurses graduating from baccalaureate programs must be able to use computers and information systems and apply data and evidence to inform practice. Those competencies are also essential for the entire nursing workforce [6]. The amount and complexity of information of nurses are expected to manage continues to increase exponentially [7]. Developing these skills has an impact on nurses' future clinical work and their ability to achieve best practice.The importance of developing information literacy skills as an undergraduate nurse has been well documented in the literature [8, 9].

With the inclusion of medical informatics and information literacy skills in required core competencies, information literacy require formal assessment [10]. Therefore, it is necessary to understand the factors influencing their information literacy and develop early and effective interventions to improve them. The purpose of this study was to investigate the information literacy among the nursing undergraduates in China, to analyze the factors potentially involved in information literacy.

## Methods

### Ethics

The Ethics Committee of the Affiliated Hospital of the Inner Mongolia Medical University approved the study protocol (approval number: 2021007). Participants were assured that it was voluntary to participate in the study and that they were free to withdraw from the study at any time. Completing and returning the questionnaire was considered as consent for participation.

### Study design

This was a cross-sectional study. Cross-sectional studies serve many purposes, and the cross-sectional design is the most relevant design when assessing the prevalence of disease, attitudes and knowledge among patients and health personnel [11].

### Setting and participants

A convenience sampling method was adopted. A questionnaire survey was conducted among nursing undergraduates. They were in their bachelor's degree program in Inner Mongolia Medical University of China at the time of survey. The connotation of each dimension of information literacy is different for students with different training levels [12]. Therefore, the surveys were conducted at four different grades. In Chinese universities, late July to August is often summer vacation, with a new semester starting in September. This study chose to survey them between October and November 2020. Seven hundred and ten students (568 females and 142 males) filled out the questionnaire.

### Outcome measurements

Information Literacy Competency Scale of the Applied Undergraduate Student (ILCSAUS).

The ILCSAUS was designed according to the Chinese cultural context by Xiaowei Wu to evaluate information literacy [13]. It comprises of a total of four dimensions with 33 items and includes 5 items for information awareness, 21 items for information skills, 3 items for information application and creation, 4 items for information security and ethics. Information awareness refers to the individual's sensitivity to information, sustained attention, insight and judgment on the value of information.Information skills include information needs planning, information acquisition, information evaluation, information organization and communication capabilities.Information application and creation refers to whether the obtained information can be applied to solve problems and whether information products can be submitted. Information security and ethics refers to the application and creation of information within the scope permitted by national laws and social civic ethics. Multilevel scoring is used (range: 1–5). A higher score indicates a higher level of information literacy. The Cronbach's alpha coefficient for the scale was 0.713.

### Data collection

The questionnaire consisted of two parts. The first part included demographic details such as gender, grade, English level (based on the National Unified Examination, with appropriate certificates), birth place, received training in literature retrieval and utilization, received training in medical statistics, and published articles. The second part was the ILCSAUS. It comprises four dimensions (information awareness, information skills, information application and creation, information security and ethics).Participants were informed that the survey was voluntary, anonymous, and the participants were assured that the confidentiality of information would be maintained. Participants were allowed to terminate the survey at any time if they wished to do so.

### Statistical analysis

SPSS 20.0 (US SPSS Inc.) was used to analyze the data. Categorical data were presented as frequencies. The Kolmogorov–Smirnov test and a histogram normal curve were used to test the normal distribution of the information literacy scores and scores in the 4 dimensions. The results of the Kolmogorov–Smirnov test showed that *p* < 0.05 and that the histogram normal curve was not concentrated and symmetrical. This indicated that the data did not follow a normal distribution and the median (inter quartile range) M (IQR) was used to describe the data. Therefore, the non-parametric Mann–Whitney U test and Kruskal–Wallis H test were used for statistical analysis. Multivariate stepwise linear regression analysis was conducted using the information literacy score as a dependent variable. The independent variables were those with *p*-values < 0.05 in univariate analyses. Two-sided *p*-values < 0.05 were considered statistically significant.

## Results

A total of 776 participants were contacted for the study. Also, 710 participants (91.5%) responded to the survey.

### Participants’ characteristics

**Fig. 1:**
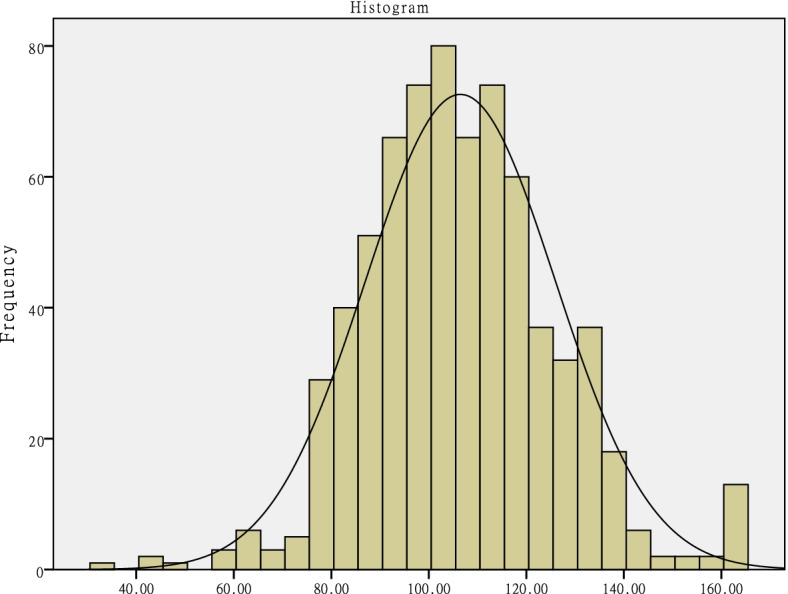
Information literacy scores normal distribution test chart. Methods: The graph of normal distribution test chart was illustrated according to Information literacy scores as abscissa and Frequency as ordinate

Socio-demographic data and information literacy scores of the participants are presented in Tables 1 and in Fig. 1. As the data did not follow a normal distribution pattern, M (IQR) was used to describe the data, and the Mann–Whitney U test and Kruskal–Wallis H test were used for analysis. The following factors were statistically significant (*p* < 0.05): grade, birth place, received training in literature retrieval and utilization, and received training in medical statistics. Gender, English level, and published articles were not associated with information literacy scores.

**Table 1:** Socio-demographic data and information literacy scores of nursing undergraduates in China (*n* = 710)

Variable	n (%)	Information literacy scores M(IQR)	Z/H	p
Gender				
Male	142(20.0)	108.00(96.00 ~ 122.25)	-1.942	.052
Female	568(80.0)	105.00(93.00 ~ 118.00)		
Grade				
First	176(24.8)	102.00(92.25 ~ 116.75)	8.690	.034*
Second	162(22.8)	107.00(95.00 ~ 120.00)		
Third	233(32.8)	105.00(92.00 ~ 117.00)		
Fourth	139(19.6)	109.00(95.00 ~ 123.00)		
English level				
CET-6	17(2.4)	110.00(98.50 ~ 128.00)	1.495	.474
CET-4	160(22.5)	106.00(95.00 ~ 119.00)		
Failed CET-4or6	533(75.1)	105.00(93.00 ~ 119.00)		
Birth place				
Country	383(53.9)	104.00(92.00 ~ 118.00)	10.751	.013*
Town	92(13)	101.50(92.00 ~ 116.00)		
County	86(12.1)	108.50(97.75 ~ 121.00)		
City	149(21)	108.00(98.00 ~ 121.00)		
Received training in literature retrieval and utilization				
Yes	155(21.8)	108.00(95.00 ~ 127.00)	-2.768	.006*
No	555(78.2)	104.00(93.00 ~ 117.00)		
Received training in medical statistics				
Yes	440(62)	108.00(95.00 ~ 120.00)	-3.354	.001*
No	270(38)	102.00(92.00 ~ 115.00)		
Published articles				
Yes	21(3)	105.00(98.00 ~ 120.00)	-.444	.657
No	689(97)	105.00(94.00 ~ 119.00)		

Abbreviations: *M(IQR)* median(Inter Quartile Range), *CET* College English Test

^*^p < 0.05

### Information literacy scores and scores in the 4 dimensions

The specifics of these results are provided in Tables 2 and Fig. 1. The data for the information literacy score and scores in the four dimensions were not normally distributed and M (IQR) was used to describe the data. The information literacy total score was 105.00 (94.00–119.00). The highest score in the four dimensions was information awareness dimension, and the item average score was 4.00 (3.80–4.40). Whereas information skills were relatively low.

**Table 2:** Information literacy scores and scores in the 4 dimensions among nursing undergraduates in China (*n* = 710)

Items	score M(IQR)	Item average score M(IQR)	Score range	Minimum	Maximum
Information awareness	20.00(19.00 ~ 22.00)	4.00(3.80 ~ 4.40)	5 ~ 25	1.00	5.00
Information skills	62.00(53.00 ~ 71.00)	2.95(2.52 ~ 3.38)	21 ~ 105	1.00	5.00
Information application and creation	9.00(8.00 ~ 11.00)	3.00(2.67 ~ 3.67)	3 ~ 15	1.00	5.00
Information security and ethics	15.00(12.00 ~ 17.00)	3.75(3.00 ~ 4.25)	4 ~ 20	1.00	5.00
Total score	105.00(94.00 ~ 119.00)	3.18(2.85 ~ 3.61)	33 ~ 165	33.00	165.00

Abbreviations: *M(IQR)* median(Inter Quartile Range)

### Factors influencing information literacy

The detailed results are given in Table 3. Multivariate stepwise linear regression analysis was conducted using the information literacy score as a dependent variable. The independent variables were those with *p*-values < 0.05 (grade, birth place, received training in literature retrieval and utilization, and received training in medical statistics) in univariate analyses. The variables that remained in the equation were: received training in medical statistics, received training in literature retrieval and utilization, and birth place.

**Table 3:** Multivariate Stepwise Linear Regression Analysis of Factors Influencing the information literacy scores among nursing undergraduates in China (*n* = 710)

Influential factor	B	SE	β	t value	*p* value
(Constant)	117.575	3.757		31.297	.000***
Received training in medical statistics	-3.823	1.532	-.095	-2.495	.013*
Received training in literature retrieval and utilization	-4.640	1.795	-.098	-2.586	.010*
Birth place	1.224	.592	.077	2.067	.039*

*F* = 7.427, *P* < 0.001, *R* = .175, *R*^2^ = .031

^*^p < 0.05, ***p < 0.001

Abbreviations: *B* partial regression coefficient, *SE* standard error, *β* standard regression coefficient

## Discussion

### Information literacy scores and scores in the 4 dimensions

This study showed that the information literacy total score was 105.00 (94.00–119.00) and item average score was 3.18 (2.85 ~ 3.61). The results of the present study are lower than Hu et al.’s [14], Lv et al.’s [15] and Chen’s study [16], and higher than Tang et al.’s study [17]. The network information has penetrated into students' study and work. Therefore, it promotes the improvement of students' information literacy ability. However, there are still phenomena of uneven and incomplete development.

The highest score was information awareness and the lowest score was Information skills in the four dimensions. Several previous studies was consistent with the results of this study [15, 16]. Some studies have revealed that nursing students have low levels of information literacy skills, which may be carried through into nursing practice if not addressed during the undergraduate years [18]. There are a number of factors that influence the development of information literacy skills and knowledge.In collaboration with academics, librarians possess the skills to assist nursing students in their quest to become information literate [19]. The findings of Tang et al. [17] have reported that the lowest score in the four dimensions was information security and ethics.

### Analysis of influencing factors of information literacy

The results showed that received training in medical statistics influenced information literacy (*p* < 0.05). Interprofessional information literacy education can generate positive learning experiences for health care professions students to increase their level understanding of research in the health care professions [20].

The results showed that received training in literature retrieval and utilization was influencing factors of information literacy (*p* < 0.05). Several previous studies have reported results similar to our findings [16, 21]. Brown et al.’s [22] study indicated that students are required to participate in a yearlong literature search project. Undergraduate nursing students who have taken the literature retrieval course have mastered the knowledge of information theory and literature retrieval skills. In the process of reviewing the literature, the more information resources a student has, the more information they reserve.

The results showed that birth place influenced information literacy (*p* < 0.05). The findings of Hu W et al. was consistent with the results of this study [14]. The level of economic development in cities is higher than in rural areas. Nursing undergraduates in cities are more likely to obtain a large amount of information resources. The city’s advanced information equipment and platforms have created conditions for nursing undergraduates to improve their information literacy. O'Farrill et al. [23] found that a good environment atmosphere is conducive to the improvement of information literacy. Therefore, in order to improve the information literacy level of nursing undergraduates, it is very important to create a good information environment and provide necessary information equipment and platforms.

## Conclusion

The innovations of this study can be summarized as the factors potentially involved in the level of information literacy of nursing undergraduates in China. Nursing undergraduates from Inner Mongolia, China were independently associated with information literacy scores regardless of whether they have received medical statistics training, they have received training in literature retrieval and utilization, and their birthplace. Special interventions to promote information literacy among nursing undergraduates need to be implemented. Learning about medical statistics, literature retrieval and utilization. During the training, provide students with various practical opportunities to exercise their ability to use information. Students born in countries and towns require particular attention. Guide students to carry out active information activities by arranging homework for information problems, introducing journals related to majors, and reading methods of professional literature, so that students can develop information skills while acquiring knowledge [24]. The conclusions of this study can provide a reference to improve the information literacy intervention strategies for nursing undergraduates.

## Research limitations and future research ideas

This study had several limitations. A convenience sampling method was adopted and participants were from the Inner Mongolia, thus limiting the generalizability of the study’s findings. The self-assessment method may limit the generalization of the findings. The study could have benefitted from a longitudinal follow-up.

## Data Availability

The datasets generated during and/or analyzed during the current study are available from the corresponding author on reasonable request.
